# SSH-YOLO: YOLOv8 improved model based on Object Detection in Complex Road Scenes

**DOI:** 10.1371/journal.pone.0343924

**Published:** 2026-04-15

**Authors:** Tenglong Ma, Yanlin Chen, Jiaqiang Li, Haisheng Yu, Chao He

**Affiliations:** College of Mechanical and Transportation Engineering, Southwest Forestry University, Kunming, China; Wuhan University of Science and Technology, CHINA

## Abstract

To address the problem of insufficient detection accuracy for dense targets, small targets and partially occluded objects in complex road scenarios, an improved object detection model, SSH-YOLO, is proposed. On the basis of YOLOv8n, the model optimizes and improves performance through a three-level collaborative architecture: 1) introduce the spatial and deep conversion (SPDConv) module in the backbone network to replace the traditional step downsampling with nonstep convolution, retain the fine-grained features of small targets, and solve the feature loss problem of low-resolution images; 2) embed the spatial and channel collaborative attention module (SCSA), through cross-scale feature fusion (SMSA) and channel weight progressive optimization (PCSA), focus on the key visible areas of the occluded target and suppress background interference such as roadside vegetation; and 3) add a new 160 × 160 resolution small object detection head, combined with the original P3‒P5 layer to form a four-level detection system, covering long-distance small targets < 32 × 32 pixels. The experimental results show that the improved model performs well on the self-built RoadScene-Complex dataset and four public datasets BDD100K: 0.729 (12.4% higher than YOLOv8n) on the RoadScene-Complex dataset mAP@0.5 (12.4% higher than YOLOv8n) and 0.868 (7.6% higher than the KITTI dataset) mAP@0.5). COCO small target subset mAP@0.5 to 0.585 (up 16.5%), and CityPersons occluded scene mAP@0.5 to 0.739 (up 22.8%). At the same time, it maintains lightweight characteristics and has an inference speed of up to 60 FPS to meet the needs of real-time on-board detection. The research results provide a balanced solution of “accuracy-speed-lightweight” for high-precision target detection in complex traffic scenarios, especially in small target and occlusion scenarios.

## Introduction

As the core task of the field of computer vision, object detection realizes the positioning and classification of targets in images or videos through algorithms and models. Its technological breakthroughs have promoted the intelligent development of autonomous driving, security monitoring, industrial quality inspection and other fields [1]. As a key application direction of this technology in the field of intelligent transportation, road object detection focuses on the accurate identification and positioning of core road elements such as vehicles, pedestrians, and traffic signs, which is the technical cornerstone of building intelligent transportation systems [2]. In practical applications, autonomous vehicles need to avoid long-distance pedestrians and judge the distance between congested vehicles through real-time object detection, and the particularity of complex road scenarios (such as low light on rainy days, dense traffic congestion in the morning and evening rush hours, and long-distance small targets on suburban roads) puts forward strict requirements for the robustness and adaptability of the detection model [3].

At present, object detection algorithms based on deep learning can be divided into two main categories: two-stage algorithms and one-stage algorithms. There are significant differences in detection accuracy, speed and scene adaptability between the two types of algorithms, and their core characteristics and applicability to road scenarios are as follows:

The two-stage object detection algorithm is represented by R-CNN [4] (region with CNN feature) and Fast R-CNN [5] The core process follows the two-step process of “candidate region generation → feature extraction and detection”. First, potential target candidate regions are generated through a selective search or area generation network (RPN), and then convolutional neural networks are used to extract features from the candidate regions. Finally, the target category determination and bounding box coordinate regression are completed. The screening mechanism of the candidate area allows it to focus on the target area, with high detection accuracy, and can accurately capture the detailed characteristics of the target. However, the additional candidate area generation step leads to high computational complexity and slow detection speed, making it difficult to meet the requirements of road scenarios with strict real-time requirements, such as in-vehicle systems.

The first-stage object detection algorithm: Without generating candidate regions, the category probability and bounding box position of the target are directly predicted on the input image through the convolutional neural network, and the detection speed is significantly better than that of the two-stage algorithm, which is the mainstream choice for real-time detection of road scenes, including SSD and YOLO series [6]. The SSD [7] algorithm: Multiscale detection is achieved by fusing convolutional feature maps at different levels—shallow feature maps (high resolution) capture detailed information of small targets, and deep feature maps (low resolution) obtain semantic information of large targets. Different levels of feature maps correspond to different receptive fields, which can be adapted to different sizes of targets in complex scenes. However, the algorithm has obvious flaws: the proportion of small target pixels is low, and it easily loses key features after multilayer convolutional downsampling, resulting in missed detection or inaccurate positioning of small objects such as “long-distance vehicles and pedestrians” in road scenarios. YOLO [8] series algorithms: With the core advantage of “balancing detection speed and accuracy”, the backbone network, feature fusion mechanism, and detection head design are continuously optimized in subsequent iterations. Among them, YOLOv8n [9], as a lightweight version in the series, has only 3.0 M parameters and has become the preferred model for automotive edge equipment because of its low computing power consumption; however, there are still three major architectural bottlenecks in complex road scenarios, which is also the core driving force of this study.

For the detection requirements of complex road scenarios, the existing architecture of YOLOv8n has the following three key bottlenecks, which directly affect its detection performance:

1) Loss of small target features: YOLOv8n’s backbone network relies on step convolution (stride = 2) and a pooling layer for downsampling to compress the feature map size and improve computational efficiency. However, this process can lead to severe dilution of fine-grained features such as edges, textures, and other fine-grained features of small targets in low-resolution feature maps (e.g., P5 layer, size 20 × 20). For example, when a bicycle is 50 m away (the pixel size is approximately 20 × 15), the missed detection rate is as high as 38%, which cannot meet the need for effective identification of small targets at long distances.
2) Insufficiently focusing on occlusion targets: Existing attention mechanisms (such as SE attention) can only optimize the weight of the channel dimension of the feature map but cannot locate the key visible areas for “partially occluded targets” in road scenes (such as pedestrians being occluded by buses and vehicles being obscured by adjacent vehicles in congestion). This limitation can easily lead to the model misjudging the occlusion feature as the target feature, which significantly improves the false detection rate and missed detection rate of the occlusion target.
3) Incomplete multiscale coverage: YOLOv8n uses only P3 (size 80 × 80), P4 (size 40 × 40), and P5 (size 20 × 20) three-level detection heads for pixel size <32 × 32 Small targets (e.g., distant pedestrians, small traffic signs) are not covered. At the same time, the existing architecture lacks a feature discrimination mechanism for “dense targets” (such as < 10 pixels between vehicles during morning and evening rush hours), which is prone to feature confusion of adjacent targets, resulting in reduced detection accuracy.

To solve the detection problems of complex road scenarios, many scholars have improved the existing object detection algorithms, but various schemes have certain limitations, making it difficult to achieve a comprehensive balance of “accuracy-speed-generalization”. Jiaqi Fan *et al* [10]. improved the accuracy and robustness of vehicle detection in complex scenarios by adjusting the size and proportion of the anchor frame of the Faster R-CNN and using an approximate joint training method. However, the proposed scheme is based on the VGG16 backbone network, which has high computational complexity and does not optimize real-time performance. Moreover, the dataset is small and lacks extreme weather (such as heavy rain and fog) data, and the generalizability under complex road conditions needs to be verified. Z Chen *et al* [11]. used the MobileNet v2 lightweight backbone network to reduce computational costs, introduced a channel attention mechanism to strengthen key feature weights, and constructed a bottom-up feature fusion structure through deconvolution, which improved the vehicle detection speed and optimized the multiscale detection accuracy. However, the proposed scheme lacks the ability to extract features from dense occlusion scenes, has a poor detection effect on small targets in complex environments such as rainy days, foggy days, and low light, and only supports single-category vehicle detection, which cannot meet the needs of multiobject detection on the road.

Wang *et al* [12]. introduced the BiFormer attention mechanism to optimize the feature extraction ability of the backbone network, proposed the Focal FasterNet block module to enhance the feature processing effect, and expanded the detection scale to 5, which improved the overall object detection performance. However, the detection accuracy of this scheme for small targets such as bicycles is still low, and it cannot effectively solve the problem of missed detection of small targets in road scenes. Rana Md *et al* [13]. added an additional convolutional layer to the backbone and head parts of YOLOv5, integrating the inception model structure with the spatial pyramid pooling layer (SPP), which enhanced the feature extraction ability and detection accuracy of the model. However, the datasets used in this scheme lack scene diversity and large annotation errors, which affects the detection accuracy. Moreover, the reasoning speed and robustness of the model in complex road scenarios are not discussed, and its practicability is limited. Hui Li [14] introduced the C2f-f module to expand the feature receptive field and retain the continuity of high-level and low-level features and carried out multiscale fusion and reconstruction of the YOLOv8 backbone network on the basis of the BiFPN principle, which effectively improved the accuracy of small object detection and the generality of the model. However, the proposed scheme does not verify the robustness of the model in scenarios such as complex occlusions and extreme weather, nor does it analyze the impact of the new module on the inference speed, which is difficult to adapt to the deployment requirements of actual road scenarios.

Future research needs to further break through the above bottlenecks to achieve more accurate, efficient, and versatile target detection algorithms for complex road scenarios while overcoming the deployment challenges caused by computing power limitations and promoting the steady development of this technology in the field of intelligent transportation. In view of the above shortcomings, this paper solves the three major bottlenecks of YOLOv8n through the collaborative design of SPDConv feature retention, SCSA feature optimization, and small target detection head scale supplementation and maintains the advantages of being lightweight and real-time [15].

## YOLOv8 detection algorithm

As a high-speed, accurate, and easy-to-use model, YOLOv8 is widely used in target detection and tracking, instance segmentation, image classification, and pose estimation [16]. Its network structure covers the input layer, the backbone network, the neck network, the detection head, and the output layer. The input layer receives image data, the backbone network extracts image features in depth through convolutional operations, the neck network fuses features of different scales and strengthens the expressive ability, the detection head determines the target category and predicts the location of the bounding box on the basis of processed features, and the output layer integrates the results of the detection head and outputs accurate detection information after postprocessing.

The YOLOv8 detector head adopts an anchor-free design to eliminate the preset anchor frame; predicts the location of the bounding box, category probability and confidence in an anchor-free decoupled way; outputs the results through the output layer of nonmaximum value suppression and confidence filtering, which is equipped with the advantages of high accuracy, fast speed, flexible deployment, and good robustness and generalization; and can detect multiscenario targets in a highly efficient and accurate way. It uses a task-alignedigner to assign samples dynamically, which improves the flexibility of positive sample frame selection and enhances detection accuracy and robustness. The loss function consists of classification loss (including BCE loss to measure the difference between the category prediction confidence and the actual label, VFL loss to focus on the positive samples and adjust the weights according to the degree of overlap) and regression loss (including CIOU loss to fit the target frame from the geometrical parameter, and DFL to convert regression into classification to optimize the location of the target and the distribution of the neighboring regions), which collaboratively helps the model learn the classification and location information.

**Fig 1:**
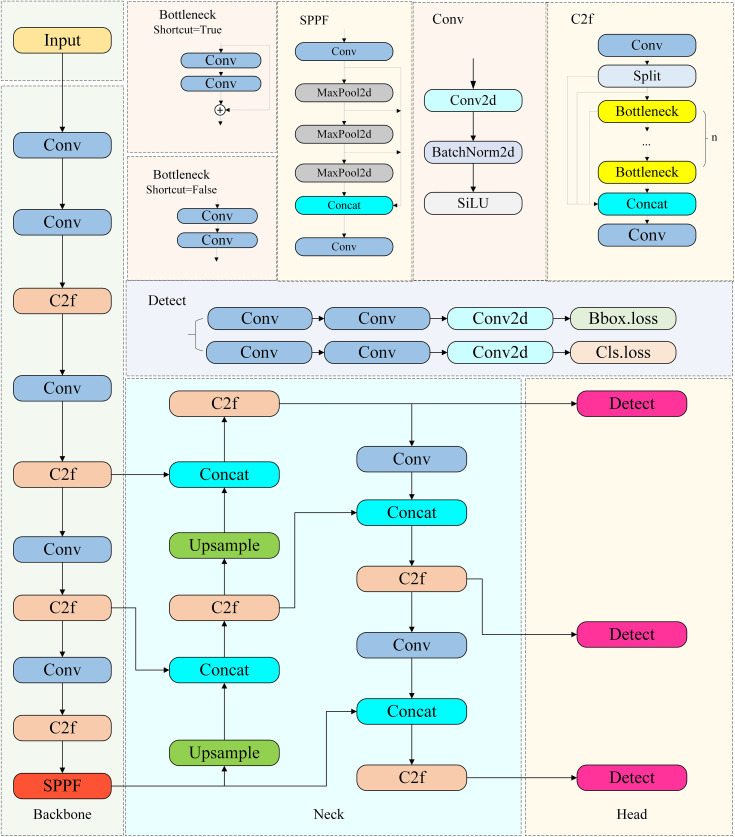
YOLOv8 network framework.

The C2f module of YOLOv8 inherits the idea of the CSP structure. It makes innovative improvements to enhance the feature fusion effect, providing better feature information for target detection. At the same time, it enhances the gradient flow, accelerates the model convergence, improves the training effect, and adapts to the overall architectural and performance requirements to push the YOLO series algorithms to increase their performance in target detection. The network framework of YOLOv8 is shown in Fig 1.

## SSH-YOLO Improved Model

There are more small targets in the collected road image samples, and the collected target feature information is easily missed, so the detection accuracy of the algorithm is low. The small target detection layer P2 of the YOLOv8 algorithm is located in the beginning stage of the network, which has a high resolution and can capture detailed information in the image more accurately. To obtain the image information in more detail, this paper adds one detection head for small targets based on the original, combined with the other three detection heads, to improve the image’s detection accuracy [17]. The SPDConv module is integrated into the backbone network of YOLOv8n to improve the image detection capability for small targets or when the image resolution is low. The spatial and channel coattention module SCSA is introduced to improve the model’s ability to focus on target features.

**Fig 2:**
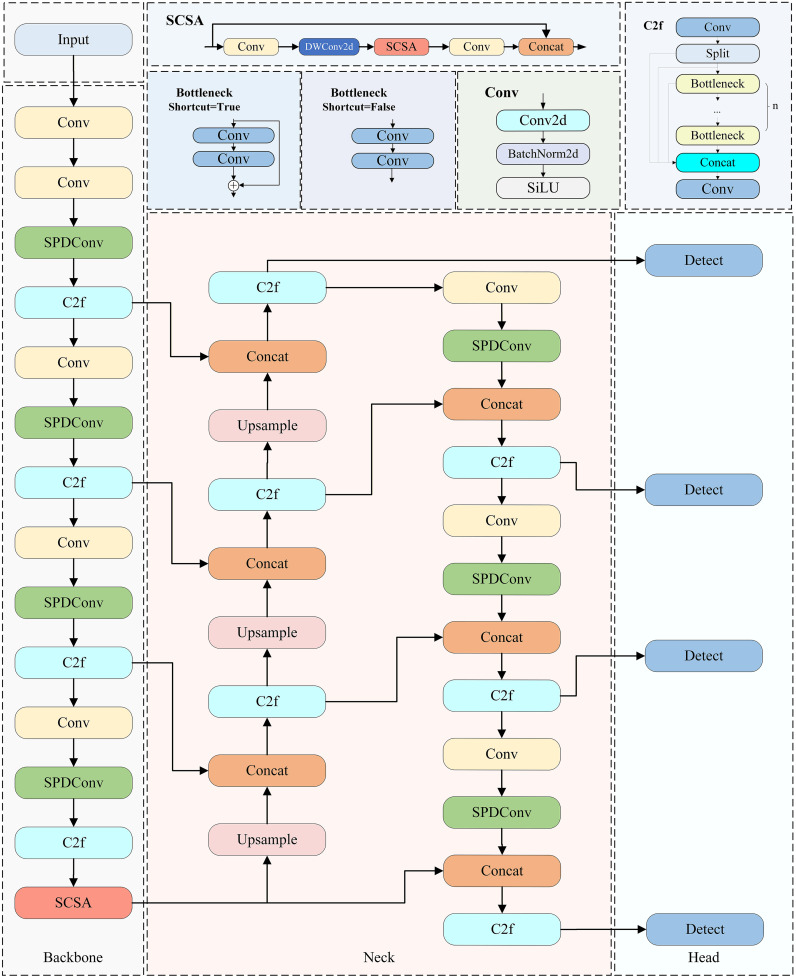
SSH-YOLO Network Framework.

With a high percentage of overlapping occlusions of detected targets in complex road driving environments, accurate localization is crucial for improving the detection of occluded targets via target detection algorithms. Improving the localization accuracy is particularly important when the target is in a complex occlusion situation. High-precision localization can enable the algorithm to more accurately lock the target’s actual position and contour information; even if the target is partially obscured, the algorithm can be based on accurate localization clues to more effectively identify and distinguish between the target and the obscured object, thus reducing the misjudgment and omission of detection due to obstruction and significantly improving the algorithm’s performance in detecting the obscured target. Thus, in complex and variable real-world scenarios, the algorithm can be more stable, reliably completing the target detection task, and providing more accurate and robust data support for subsequent analysis and decision-making [18]. The SSH-YOLO network structure is shown in Fig 2.

### The SPDConv Module

The YOLO algorithm is based on a deep convolutional neural network for object detection. Convolutional neural networks have achieved remarkable success in computer vision tasks such as image classification and object detection, but their performance can be catastrophic when the image resolution is low or when the object is small [19]. This is due to flaws in the common design architecture of existing CNNs, namely, the use of convolutional steps and/or pooling layers, which leads to the loss of fine-grained information and less efficient learning of feature representations, and the resolution of the image is strongly related to the accuracy of the detection results.

In real road scenes, the external environment in which the vehicle is located is complex and changeable, and complex scene factors such as lighting changes, partial occlusion, scale changes, background interference, and adverse weather conditions present many difficulties and challenges for object detection tasks. On this basis, SPD-Conv, which consists of an SPD layer and a nonstrided-Conv layer, was introduced [20].

SPD-Conv (spatially separated and deformable convolution) is a convolution operation optimized for low-resolution images and small object detection, and its innovation is that it better preserves the details of the image by avoiding convolution and pooling operations with a step size greater than 1 in traditional convolution. SPD-Conv divides the input image into multiple subgraphs, merges these subgraphs via feature fusion, and finally further processes it through a convolutional layer with a step size of 1 to obtain a finer feature representation. This structure is particularly suitable for processing low-resolution images and can improve the model’s performance in tasks such as small object detection [21].

**Fig 3:**
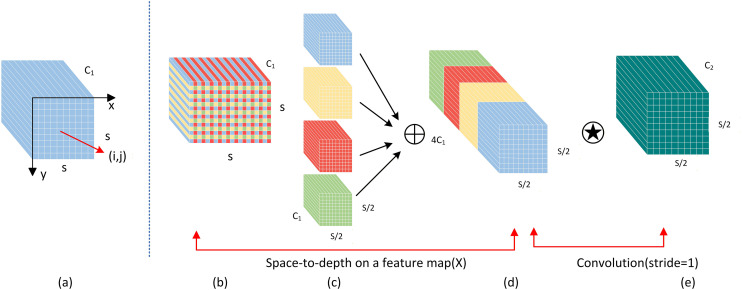
SPDConv Network Framework.


As shown in Fig 3, SPDConv at scale = 2, a represents the standard feature map, b is the space-to-depth operation, c is an example of the depth increase of the resulting feature map, d represents the nonstep convolutional layer applied after the SPD operation, and e represents the output feature map after step 1 convolution, which maintains spatial resolution but changes the depth dimension. As shown in the figure, starting from the middle feature diagram X of any size S × S × , C-1., a series of subfeature diagrams are first sliced out, and the principle of the splitting formula is as follows:

$$f_{\text{0},\text{0}}=X[\text{0}:S:scale,\text{0}:S:scale],f_{\text{1},\text{0}}=X[\text{1}:S:scale,\text{0}:S:scale],\ldots ,$$

$$f_{scale-\text{1},\text{0}}=X[scale-\text{1}:S:scale,\text{0}:S:scale];f_{\text{0},\text{1}}=X[\text{0}:S:scale,\text{1}:S:scale],f_{\text{1},\text{1}},\ldots ,$$

$$f_{scale-\text{1},\text{1}}=X[scale-\text{1}:S:scale,\text{0}:\text{1}:scale];f_{\text{0},scale-\text{1}}=X[\text{0}:S:scale,scale-\text{1}:S:scale],f_{\text{1},scale-\text{1}},f_{\text{1},scale-\text{1}},\ldots ,$$

$$f_{scale-\text{1},scale-\text{1}}=X[scale-\text{1}:S:scale,scale-\text{1}:S:scale].$$

For any (original) feature map X, the subgraph $f_{x,y}$ consists of all the elements X(i,j) that satisfy i + x and j + y and can be divisible by scale. Therefore, each subgraph downsamples X scale by a factor of two. In the figure, (a), (b), and (c) present an example of scale = 2, in which case we obtain four subgraphs, $f_{\text{0},\text{0}}$, $f_{\text{1},\text{0}}$, $f_{\text{0},\text{1}}$, and $f_{\text{1},\text{1}}$, each subgraph is shaped like ($\frac{S}{\text{2}}$, $\frac{S}{\text{2}}$, and C_1_), and X is downsampled 2 times. Next, these subfeature maps are connected along the channel dimension to obtain a feature map, $X^{\prime }$, whose spatial dimension is reduced by scale, and the channel dimension is increased by ${scale}^{\text{2}}$. Then, the SPDtransforms the feature map X(S,S,C_1_) into an intermediate feature map, $X^{\prime }\left(\frac{S}{scale},\frac{S}{scale},{scale}^{\text{2}}C_{\text{1}}\right)$, and finally connects anonstep convolution (step size 1) to retain as much discriminant feature information as possible.

A detailed analysis of the structure and principle of SPD-Conv reveals that it effectively improves the performance of the model by controlling the step size of convolution operations, fusing features of different scales, and utilizing multichannel convolution, especially in the case of low-resolution input and small object detection, avoiding the information loss caused by traditional convolution and pooling.

### SCSA attention mechanism

In complex road scenarios, the core challenges of object detection are the sparseness of small target features (such as the proportion of pixels of long-distance pedestrians <3%) and the semantic confusion of occlusion targets (such as the overlap between occlusion and target features in vehicle congestion). Existing single-dimensional attention mechanisms (such as channel domain SE and spatial domain SAM) cannot solve two types of problems at the same time: Zhang *et al* [22]. clearly pointed out that autonomous driving scenarios need to achieve “weak feature enhancement - key area localization - Background suppression”, which provides the core theoretical framework for the design of the SCSA Channel Synergistic Attention) module.

#### Design background and core basis.

The particularity of road scenes puts forward three requirements for attention mechanisms:

Multi-scale adaptability: Small and large targets coexist, and the range of the receptive field needs to be dynamically adjusted.

Occlusion robustness: The effective feature fragmentation of partial occlusion targets (such as pedestrians being occupied by buses) requires precise positioning of visible areas.

Lightweight constraints: The computing power of the vehicle edge device is limited, and the number of attention module parameters needs to be controlled within 0.5M.

The traditional attention mechanism has obvious limitations: the SE module only optimizes the channel weight, and the spatial position of small targets is not captured enough. Although CBAM combines space-channel, it uses a fixed 3 × 3 convolutional kernel, and the receptive field does not match the small target size. Tao *et al* [23]. although the RFAConv proposed by the sensory field attention improves the detection accuracy of industrial small objects, it is not adaptable to the dynamic occlusion of the road for static scene design.

To this end, SCSA adopts a two-level linkage architecture of “Multi-Scale Semantic Capture (SMSA)-Channel Weight Progressive Calibration (PCSA)”: SMSA solves the problem of multi-scale and occlusion positioning in the spatial domain, and PCSA realizes the enhancement of weak features in the channel domain and semantic interference filtering, which synergistically meet the special needs of road scenarios.

**Fig 4:**
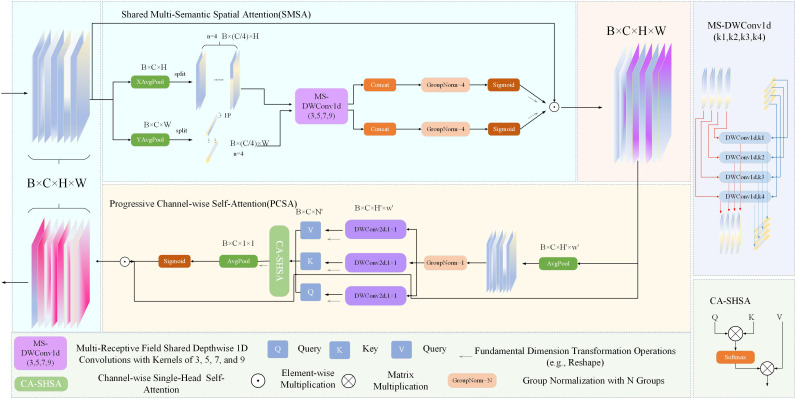
SCSA network framework.

Fig 4 presents a visual schematic of spatial and channel cooperative attention (SCSA). The symbol B represents the batch size, C represents the number of channels, and H and W correspond to the height and width of the feature map, respectively. The variable n specifies the number of groups in which the subfeatures are divided, and 1P refers to a single pixel [24].

The SCSA spatial and channel collaborative attention mechanism is composed of two main parts, namely, shareable semantic spatial attention (SMSA) and progressive channelwise self-attention (PCSA). These two parts work together to bring unique advantages to the model’s feature processing. Among them, the SMSA is used to integrate multisemantic information, and the progressive compression strategy is used to inject discriminative space priors into the channel self-attention of the PCSA, effectively recalibrating the channel. The robust feature interaction based on the self-attention mechanism in the PCSA further alleviates the multisemantic information differences between different subfeatures in the SMSA, specifically:

SMSA: Multiscale DWConv1d (Depthwise 1D Convolutions) is used to extract spatial information at different semantic levels from four independent subfeatures. GroupNom is used to accelerate model convergence while avoiding the problem of introducing batch noise and semantic information leakage between subfeatures.

PCSA: This method minimizes computational complexity by combining progressive compression and channel-specific self-attention mechanisms while preserving spatial priors within the SMSA. In addition, the PCSA further explores channel-level similarities by using the self-attention mechanism, thereby reducing semantic differences between different subfeatures.

For a given input X, the implementation of SCSA is as follows:

SMSA: First, it is decomposed along the H and W dimensions, and global average pooling is carried out to establish two one-way one-dimensional sequence structures. To learn different spatial distributions and contextual relationships, the feature set is divided into K independent subfeatures of the same size ($X^{i}$. represents the ith subfeature, where *i* ∈[1,*K*]).


$$\overset{i}{\underset{H}{X}}=X_{H}\left[:,(i-\text{1})\times \frac{C}{K}:i\times \frac{C}{K},:\right]$$

$$\overset{i}{\underset{W}{X}}=X_{W}\left[:,(i-\text{1})\times \frac{C}{K}:i\times \frac{C}{K},:\right]$$ (1)

The data are processed via multiscale DWConv1d. The multiscale mechanism is used to capture the different semantic spatial structures within each subfeature more efficiently. To solve the finite receptive domain caused by feature decomposition and one-dimensional convolution, after DWConv1d, lightweight shared convolution is used for feature alignment. Finally, the different semantic subfeatures are aggregated and normalized via GroupNorm, and then the sigmoid activation function is used and multiplied by X to generate spatial attention.


$${Attn}_{H}=\sigma (\overset{K}{\underset{H}{GN}}(Concat\left(\overset{\text{1}}{\underset{H}{\tilde{x}}},\overset{\text{2}}{\underset{H}{\tilde{x}}},\ldots ,\overset{K}{\underset{H}{\tilde{x}}}\right)))$$

$${Attn}_{W}=\sigma (\overset{K}{\underset{W}{GN}}(Concat\left(\overset{\text{1}}{\underset{W}{\tilde{x}}},\overset{\text{2}}{\underset{W}{\tilde{x}}},\ldots ,\overset{K}{\underset{W}{\tilde{x}}}\right)))$$

$$\text{SMSA}(\text{X})=\text{XS}={Attn}_{H}\times {Attn}_{W}\times X$$ (2)

PCSA: The feature Xs processed by the SMSA are first averaged and then normalized via GroupNorm. Generate Q, K, V by using a multibranch DWConv. and through self-attention aggregation. Finally, an average pooling layer and sigmoid activation function are used to generate attention graphs ($F_{proj}(\cdot )$ indicates the generation of linear projections of Q, K and V).


$$X_{P}=\overset{(H,W)\to \left(H^{\prime },W^{\prime }\right)}{\underset{\left(K,K\right)}{Pool}}\left(X_{S}\right)$$

$$F_{proj}=\overset{C\to C}{\underset{\left(\text{1},\text{1}\right)}{\text{DWConv1d}}}$$

$$Q=\overset{Q}{\underset{proj}{F}},K=\overset{K}{\underset{proj}{F}}\left(X_{P}\right),V=\overset{V}{\underset{proj}{F}}\left(X_{P}\right)$$

$$X_{attn}=Attn(Q,K,V)=Softmax\left(\frac{{QK}^{T}}{\sqrt{C}}\right)V$$

$$\text{PCSA}({\text{X}}_{S})={\text{X}}_{C}={\text{X}}_{S}\times \sigma (\overset{\left(H^{\prime },W^{\prime }\right)\to (\text{1},\text{1})}{\underset{\left(H^{\prime },W^{\prime }\right)}{Pool}}\left(X_{attn}\right))$$ (3)

SCSA enables features of different scales to complement each other through cross-scale feature fusion, enhancing the representation ability of small targets. Especially in small object detection, low-level features (rich in detail) and high-level features (rich in semantic information) can be effectively combined to improve detection accuracy. For partially occluded targets, the SCSA can automatically identify and focus on key parts of the occluded area to avoid object detection failures due to occlusion. Through cross-scale information interaction, the potential features of the occluded part can be recovered, thereby improving the robustness of object detection. It is also possible to suppress the interference of background information by selectively focusing on the characteristics associated with the target.

SCSA is implemented in a well-organized manner with high interpretability, can efficiently capture important information between different channels, and can finally output a double-optimized feature map.

### A Small Object Detection Layer

Small targets such as bicycles, motorcycles, pedestrians, and small animals are easily encountered while driving, and small-scale targets are generally referred to as long distances. In the actual road driving process, the road environment, such as curves and slopes, roadside facilities and objects, bad weather, and traffic conditions, such as dense queuing of vehicles and the behavior of traffic participants, lead to the emergence of small targets and obscuring situations.

In road target detection tasks, the center position information of the target is essential for accurate detection and subsequent processing. When the center position of a target is ambiguous or uncertain, it may be because the target is partially occluded or inaccurately detected. For example, a pedestrian’s lower body is obscured by a billboard, resulting in uncertainty about the location of the lower part of the bounding box. At this point, the detection results for this pedestrian are relatively less reliable. On the other hand, a target with a precise center location is more likely to have its complete features accurately extracted, so in the case of multitarget detection, this type of target will be prioritized for further processing [25].

The center position of the bounding box is a key parameter in YOLO’s output. Vehicles or pedestrians with well-defined center locations are more likely to be accurately classified and localized for small road targets. In the YOLOv8 model, target recognition relies solely on the features extracted from the P3, P4, and P5 levels, which correspond to 80 × 80, 40 × 40, and 20 × 20 detector heads, respectively, in order of size. Small targets. For this reason, the initial network architecture can be supplemented with an additional detection layer that outputs 160 × 160 feature maps, which can be used to identify small targets on the road exceptionally effectively with the output of smaller-scale features [26].

**Fig 5:**
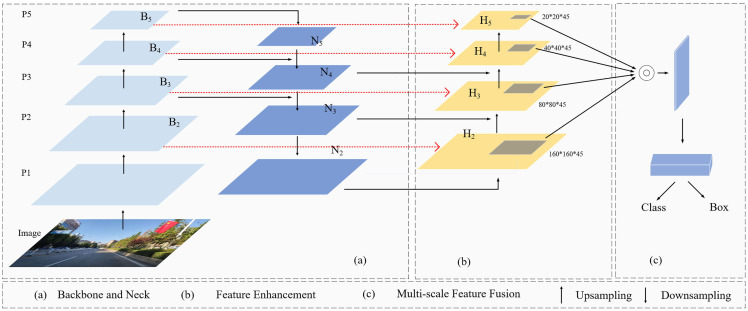
Schematic diagram of the multidetection head network structure.

Fig 5 shows a schematic of a multidetection head network structure after adding a small target detection layer to the YOLOv8 model.

## Experimental procedure

### Dataset

To verify the detection performance for complex road scenarios, this paper constructs the RoadScene-Complex dataset(https://doi.org/10.6084/m9.figshare.30869933), and the specific information is as follows:

1) Collection background: Covering various types of roads in Kunming, Yuxi and Dali Bai Autonomous Prefecture in Yunnan Province from December 2023 to September 2024, including highways (Kunshi Expressway, Dali Expressway), urban trunk roads (Kunming Beijing Road, Yuxi Hongta Avenue), and rural roads (roads around Dali Xizhou Ancient Town), covering 4 types of weather: sunny, rainy, foggy, and night, and 3 time periods of morning peak, evening peak, and flat peak.
2) Acquisition equipment: two types of hardware are used to ensure data diversity: 1) a vehicle dash cam (360 intelligent cloud mirrors S800, resolution 1920 × 1080, frame rate 30 fps), which is installed on the front windshield of the car; and 2) a handheld high-definition camera (SONY Alpha 7RII., resolution 3840 × 2160, frame rate 24 fps), with shooting angles covering head-up and overhead views.
3) Data annotation: Using the LabelImg 1.8.6 tool, the annotation rules follow the PASCAL VOC format, and the annotation targets include 6 types of road core elements: car, person, truck, bicycle, bus, and motorcycle.
4) Data statistics: A total of 7676 images (JPG lossless compression format) are marked, and the total number of labeling boxes is 62943. According to the target pixel size, small targets (<32 × 32) account for 35%, medium targets (32 × 32 ~ 96 × 96) account for 48%, and large targets (>96 × 96) account for 17%. The subsets are divided according to the complexity of the scene: the high-occlusion subset (occlusion rate >50%) accounts for 50%, the high-density subset (target spacing < 20 pixels) accounts for 40% (3070 photos), and the conventional subset accounts for 10% (768 photos).
5) Randomly divide the dataset into a training set, a validation set, and a test set at a ratio of 8:1:1 and ensure that the target size distribution and scene type proportion of each subset are consistent with those of the original dataset to avoid data skewness.

**Fig 6:**
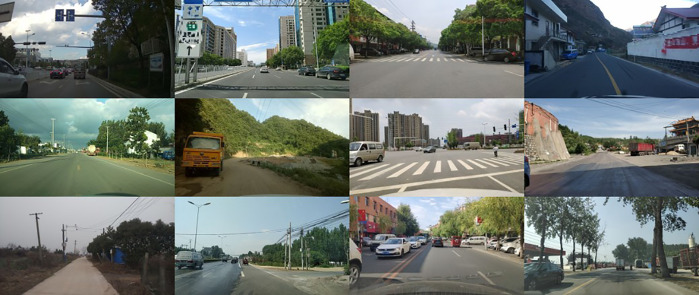
Self-Constructed Road Dataset.

To further verify the generalization ability and multiscenario adaptability of the model, four public datasets were introduced for supplementary experiments (Fig 6):

COCO: As a general object detection benchmark, its small target subset and dense crowd scenarios can effectively evaluate the model’s ability to detect small targets and densely distributed targets [27].

CityPersons focuses on urban pedestrian detection, where high-occlusion samples account for approximately 30%, and dense pedestrian scenes account for 45%. This dataset is suitable for verifying the model’s robustness in complex occlusions and dense crowds [28].

BDD100K: A dataset of real driving scenarios, including 10 weather conditions, such as sunny, rainy, foggy, and night, as well as multiple scenarios, such as urban roads, highways, and rural roads, is used to test the model’s generalization ability in lighting changes and complex environments [29].

KITTI: A visual benchmark dataset focusing on autonomous driving scenarios, the data are collected from real road scenes, and easy/moderate/hard three-level difficulty division is set on the basis of the target size and degree of occlusion, which is suitable for evaluating the detection robustness of the model in complex occlusion scenes, especially in challenging weather conditions such as low light and rainy days [30].

### Experimental environment

The experimental software environments used in this study are Windows 11, Python 3.10, and PyTorch 1.11.0. The hardware configuration includes an NVIDIA GeForce RTX 4090 GPU and Intel(R) Core(TM)i9-13900K 3.00 GHz. A momentum factor of 0.937 is introduced during the optimization process, and the input images are normalized to a uniform 640 × 640 resolution. The model training lasts for 300 epochs, and the initial learning rate is set to 0.01, while a weight decay coefficient of 0.0005 is used for the regularization constraints.

### Experimental environment

To evaluate the overall performance of the model, precision (P), recall (R), mean average precision (mAP), and the number of model parameters (Params) are used as the evaluation indices. The specific formulas for these evaluation metrics are as follows:


$$P=\frac{TP}{TP+FP}$$ (4)

$$R=\frac{TP}{TP+FN}$$ (5)

$$AP=\int_{\text{0}}^{\text{1}}P(R)dR$$ (6)

$$mAP=\frac{\text{1}}{N}\sum_{i=\text{1}}^{N}{AP}_{i}$$ (7)

where: TP (truth positive): positive class, actual positive class, i.e., correct prediction; TN (truth negative): negative class, actual negative class, i.e., correct prediction; FP (false positive): positive class, actual negative class, i.e., incorrect prediction; FN (false negative): negative class, actual positive class, i.e., incorrect prediction; predict correctly (positive or negative), i.e., T, otherwise P; predict positive class, i.e., P (correct or not), otherwise harmful class N; predict positive class, i.e., P (correct or not), otherwise negative class, i.e., correct or not), otherwise negative class N. Positive class, i.e., incorrect prediction; predict correctly (regardless of positive or negative), i.e., T, otherwise P; and predict positive class, i.e., P (regardless of correctness), otherwise negative class N.

The precision (P) reflects the proportion of actual positive cases among the positive cases determined by the classifier. Recall that (R) represents the proportion of correctly determined positive cases among the total positive cases. Recall that (R) reflects the proportion of correctly determined positive cases to the total number of positive instances. AP is the area under the P-R curve; usually, the better a classifier is, the higher the AP value. The mAP is the average of the APSs of multiple categories. The parameters refer to the total number of parameters to be trained in the model, reflecting the size and complexity of the model. Inference speed (FPS) refers to the average time it takes for the model to continuously detect 1000 images of the test set and is an important indicator for calculating the inference speed of the model.

## Experimental results and analysis

### Univariate improvement program comparison experiment

#### Validity verification of the backbone network.

To more intuitively and fully demonstrate the outstanding advantages and practical results of incorporating SPDConv into the backbone network, we deliberately introduce the variable kernel convolution AkConv and the feeling of wild attention convolution RFAConv. Moreover, we select the popular mainstream backbone network to conduct a comparative investigation with it in parallel. The corresponding experimental results are shown in Table 1 (using a self-built road dataset for validation).

**Table 1:** Comparison results.

Model	P%	R%	mAP@0.5/%	mAP@0.5:0.95/%
**SPDConv**	0.717	0.652	0.723	0.52
**SCConv**	0.628	0.53	0.583	0.383
**RePViTBlock**	0.632	0.53	0.581	0.382
**RAFConv**	0.648	0.553	0.6	0.399
**AkConv**	0.672	0.535	0.593	0.391

The experimental results in Table 1 indicate that SPDConv achieves whole index leadership through structured parameter decomposition or dynamic convolution, which is suitable for high-precision scenarios. RAFConv adopts an area-aware feature enhancement mechanism, but the recall rate is low (R% = 0.553), which needs to be cooptimized in combination with the other models. AkConv has a high precision rate (P% = 0.672) but is weak for dense target detection (R% = 0.535). The performance of the SCConv and RePViTBlock convolutional networks is significantly lower than that of the other models, and they are not recommended without special needs.

#### Validity verification of the attention mechanism.

In this paper, the SCSA attention mechanism is introduced into the YOLOv8 network structure to improve the accuracy of road target detection. Compared with other prevalent attention mechanism modules, the experimental comparison results confirm the apparent superiority of the SCSA attention mechanism module in terms of performance. The specific results are shown in Table 2 (using a self-built road dataset for validation).

**Table 2:** Comparison results.

Model	P%	R%	mAP@0.5/%	mAP@0.5:0.95/%
**SCSA**	0.636	0.55	0.6	0.396
**EMA**	0.657	0.537	0.59	0.389
**SEAttention**	0.341	0.531	0.592	0.391
**MSCA**	0.638	0.527	0.587	0.392

On the basis of the experimental results in Table 2, the SCSA attention mechanism and the other three modules yield the best results in terms of the complete index results, and the accuracy rate is significantly higher than that of the other three modules; the EMA module has a higher accuracy rate, but the performance is insufficient at high thresholds; the SEAttention module has a slightly better performance at high thresholds, but the misdetection rate is exceptionally high; and the MSCA module has various indices close to the baseline, with no outstanding advantages.

### SSH-YOLO improved the performance verification of the model

#### Ablation experiments.

To prove the accuracy performance improvement of the original YOLOv8 model by using the SPDConv module, SCSA attention mechanism module and small-target detection layer module, we use the self-constructed road dataset and add the improvement schemes one by one, and the improvement scheme of adding the SPDConv module based on the original model YOLOv8 is labeled A; the SCSA attention mechanism module B; and the small-target detection layer C. Ablation experiments are arranged to verify the results in Table 3. The design of the ablation experiment is arranged for comparative verification, and the results are shown in Table 3.

**Table 3:** Ablation Experiments.

Model	Improvement Scheme	P%	R%	mAP@0.5/%	mAP@0.5:0.95/%
**YOLOv8n**	——	0.671	0.535	0.599	0.394
**YOLOv8n**	+A	0.717	0.652	0.723	0.52
**YOLOv8n**	+B	0.636	0.55	0.6	0.396
**YOLOv8n**	+C	0.66	0.568	0.626	0.43
**YOLOv8n**	+A + B	0.71	0.67	0.718	0.525
**YOLOv8n**	+A + B + C	0.711	0.681	0.729	0.538

According to the results of the experimental data in Table 3, the addition of the SPDConv convolution module significantly outperforms the original model YOLOv8; when the SCSA attention mechanism is used alone, the expected results are poor, and it needs to be used in combination with the convolution module to reflect the value of its application. The addition of the small-target detector head slightly improves the detection results, and the recall significantly increases when SPDConv is optimized in harmonious collaboration with the SCSA. It is further enhanced when SPDConv, the SCSA, and a small target detection head are combined; the detection results are optimal, especially at high thresholds.

#### Comparison results of the models.

The optimization comparison experiments were conducted using the self-constructed road dataset before and after the algorithm improvement, and their comparison details are listed in Table 4.

**Table 4:** AP.

Class	YOLOv8n	SSH-YOLO
**Car**	0.811	0.913
**Person**	0.544	0.767
**Truck**	0.644	0.737
**Bicycle**	0.29	0.479
**Bus**	0.64	0.738
**Motorcycle**	0.666	0.739

The experimental results in Table 4 show that SSH-YOLO optimizes vehicle feature extraction better than YOLOv8 does. It is also more robust to pedestrians in multipose and occlusion scenarios and optimizes the ability to detect large and small targets.

The following graph compares the accuracy curves of the YOLOv8 and SSH-YOLO models.

**Fig 7:**
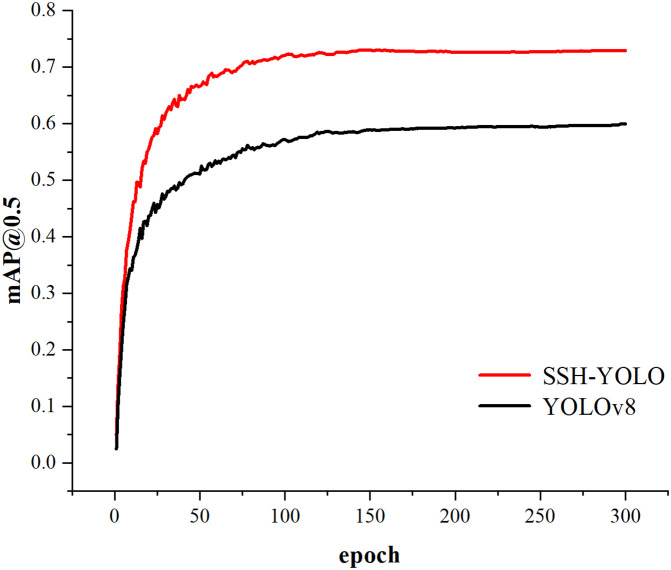
Model Comparison mAP@0.5 Curve Graph.

As shown in Fig 7. At the beginning of training, the mAP@0.5 value of the SSH-YOLO model increased rapidly, quickly surpassing that of the YOLOv8n model. In the later stage of training, the mAP@0.5 value of the SSH-YOLO model stabilized at a high level of approximately 0.7. Moreover, the YOLOv8n model also improved but finally stabilized at approximately 0.6, which was lower than that of the SSH-YOLO model. In general, the SSH-YOLO model outperforms the YOLOv8n model in terms of the mAP@0.5 index of the training task.

**Fig 8:**
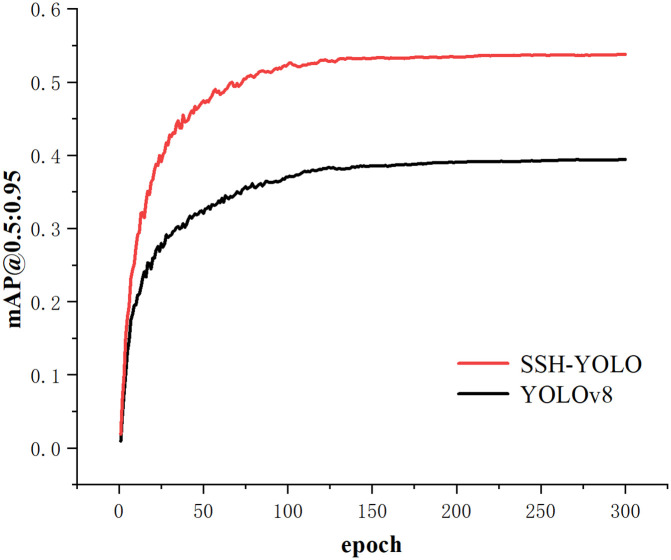
Model Comparison mAP@0.5:0.95 Curve Graph.

As shown in Fig 8. In the early stages of training, the mAP@0.5:0.95 value of the SSH-YOLO model quickly improved and surpassed that of the YOLOv8n model. In the later stages, SSH-YOLO stabilizes at a level above approximately 0.5, whereas the YOLOv8n model eventually stabilizes at approximately 0.4. Overall, the SSH-YOLO model outperforms the YOLOv8n model in terms of this evaluation metric.

**Fig 9:**
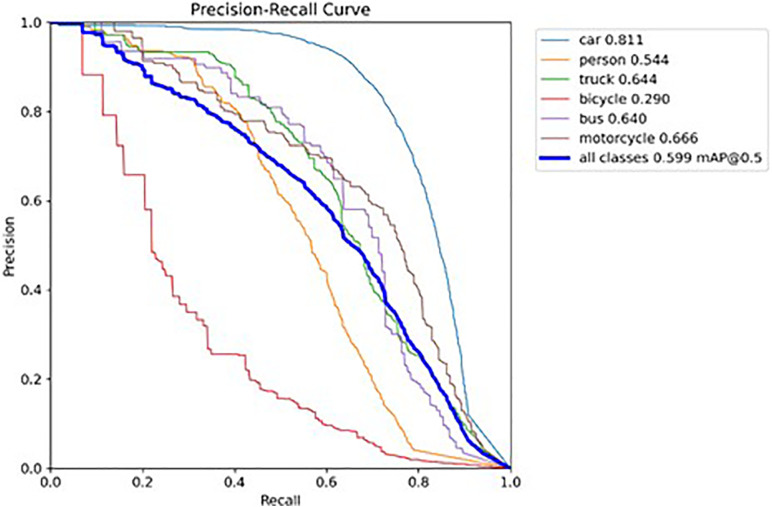
P-R Curve of YOLOv8 Model.

**Fig 10:**
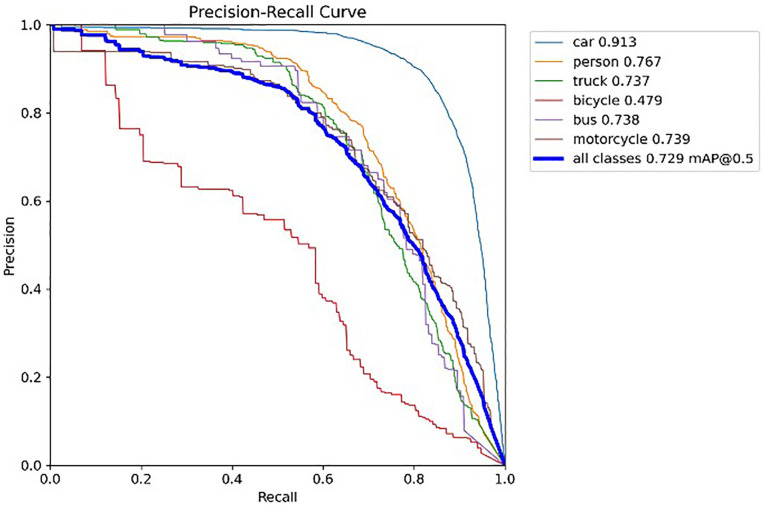
P-R Curve of SSH-YOLO Model.

According to the results of the P-R curve plots in Figs 9 and 10, the SSH-YOLO model has a more comprehensive performance. Its P-R curve tends to be closer to the upper-right region of the coordinate plot, with a larger coverage area under the curve. This feature indicates that the proposed method has higher recall and precision, reflecting the model’s superior performance.

#### Visualization analysis.

**Fig 11:**
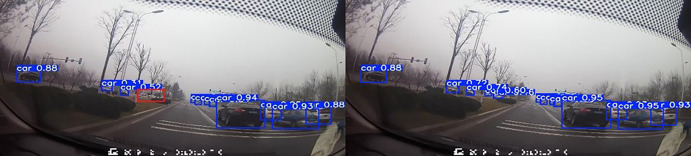
Partially Occluded Scenes.

**Fig 12:**
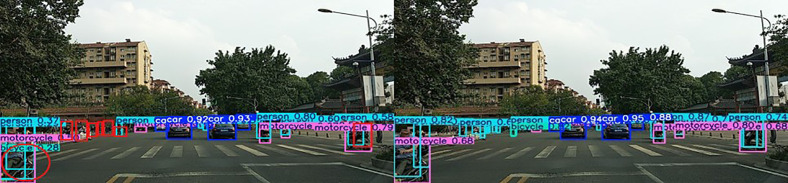
Dense targeting scenarios.

**Fig 13:**
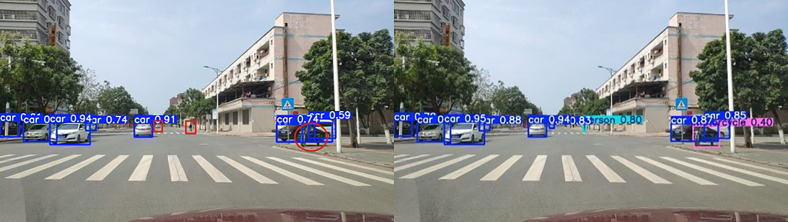
Small target scenarios.

To effectively verify the improved model’s performance in complex road scenarios, traffic scenarios in different situations are selected to compare the differences between the models, as shown in Figs 11–13 (Self-constructed Road Datasets).

When the confidence level of the detected target is high, the detected target is accurate, the exact details are captured more carefully, and the learning ability is better.

#### Heatmap visualization.

**Fig 14:**
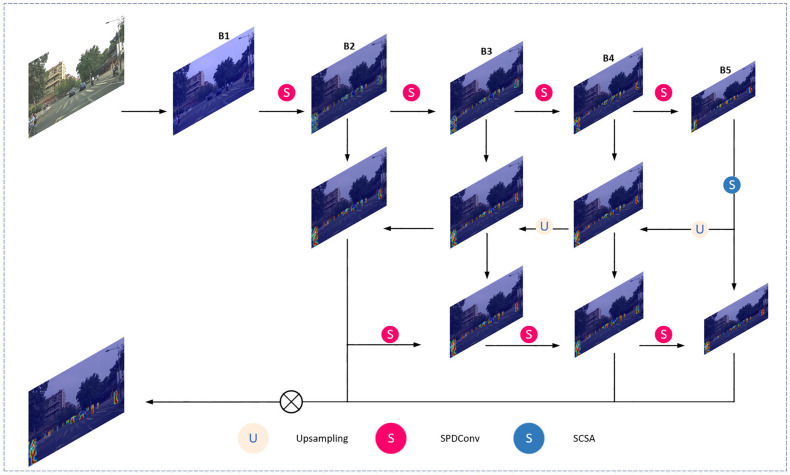
Visualization of feature extraction.

**Fig 15:**
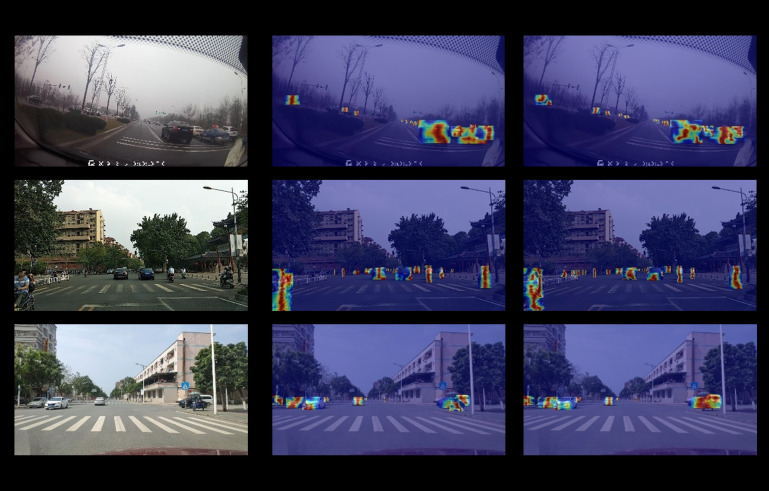
Visualization of the heatmap.

To highlight the spatial resolution, cross-scale information extraction and fusion and information reasoning capabilities of SSH-YOLO for dense targets, small targets and partially occluded targets in complex road scenarios, the Grad-CAM method is used to generate a feature heatmap (the gradient weight of the target category on the feature map is calculated via backpropagation, and the bright area represents the key feature areas of interest of the model), and the feature focusing abilities of YOLOv8n and SSH-YOLO are compared, as shown in Figs 14 and 15.

A comparison of heatmaps reveals that the heatmap of SSH-YOLO focuses more on the key areas of the target (such as the structural characteristics of small targets and the visible part of the target that occludes the target), whereas the heatmap of YOLOv8n is easily disturbed by background and occlusion, which proves the effectiveness of the improved module.

#### Comparison results of the models.

Comparative experiments with different models are conducted to more intuitively reflect the changes in SSH-YOLO performance enhancement. The experimental results are compared in Table 5.

**Table 5:** Comparison results.

Model	P%	R%	mAP @0.5/%	mAP @0.5:0.95/%	Parameters /M	FPS
**SSH-YOLO**	0.711	0.681	0.729	0.538	3.3	60
**RTDETR**	0.65	0.562	0.608	0.403	31	20
**YOLOv3-tiny**	0.657	0.43	0.487	0.301	12.1	40
**YOLOv5n**	0.662	0.526	0.587	0.384	2.5	65
**YOLOv6n**	0.628	0.549	0.578	0.38	2.6	64
**YOLOv8n**	0.671	0.535	0.599	0.394	3.0	61
**YOLOv9c**	0.75	0.618	0.702	0.507	25	31
**YOLOv10n**	0.635	0.528	0.579	0.384	4.2	56
**YOLOv11n**	0.647	0.533	0.586	0.383	2.5	65
**YOLOv12n**	0.697	0.505	0.589	0.389	2.5	65

Table 5 compares the performance of different object detection models: SSH-YOLO has the best detection accuracy, with an accuracy of 0.711, a recall rate of 0.681, an mAP@0.5 of 0.729, and only 3.3 M parameters, which has significant comprehensive advantages. In the lightweight model, the parameters of YOLOv5n, YOLOv11n and YOLOv12n are all 2.5 M, and the mAP of YOLOv5n is significant at 0.5 is 0.587, which achieves a good balance between accuracy and computational cost. On the other hand, for RTDETR, although the number of parameters is as high as 31 M, the accuracy is only 0.65, the detection efficiency is insufficient: mAP@0.5:0.95 is 0.403, and the same YOLOv9c also has the problems of too many parameters and too low accuracy. The overall data show that SSH-YOLO can be preferable when the target is dense and the traffic complex scenario has a high demand for accuracy, whereas in the edge deployment scenario with limited resources, lightweight models such as YOLOv5n are more applicable for development, which provides a quantitative reference for the scenario-based application of the model. The FPS is maintained at 60, which proves the balance between the accuracy and speed of the three-level collaborative design.

## Verification and analysis of the generalization performance of multiple datasets

### Verification of the generalization performance of multiple datasets

YOLOv12: The latest general detection model, especially the introduction of a feature enhancement module in small target detection.

RTDETR: Transformer global modeling capability, which shows better detection accuracy and robustness than traditional CNN detectors in occluded and dense target scenes.

The verification results of generalization performance of multiple models for multiple datasets are shown in Table 6–9.

**Table 6:** Comparison results.

Model	P%	R%	mAP @0.5/%	mAP @0.5:0.95/%	Parameters /M	FPS
**YOLOv8n**	0.673	0.45	0.502	0.297	3.0	61
**YOLOv12n**	0.619	0.434	0.483	0.29	2.5	65
**RTDETR**	0.569	0.463	0.495	0.302	31.9	20
**SSH-YOLO**	0.671	0.534	0.585	0.363	3.3	60

**Table 7:** Comparison results.

Model	P%	R%	mAP @0.5/%	mAP @0.5:0.95/%	Parameters /M	FPS
**YOLOv8n**	0.778	0.516	0.602	0.373	3.0	61
**YOLOv12n**	0.805	0.491	0.594	0.364	2.5	65
**RTDETR**	0.67	0.534	0.607	0.361	31.9	20
**SSH-YOLO**	0.825	0.641	0.739	0.488	3.3	60

**Table 8:** Comparison results.

Model	P%	R%	mAP @0.5/%	mAP @0.5:0.95/%	Parameters /M	FPS
**YOLOv8n**	0.56	0.361	0.358	0.193	3.0	61
**YOLOv12n**	0.461	0.322	0.341	0.184	2.5	65
**RTDETR**	0.488	0.292	0.287	0.14	32.0	20
**SSH-YOLO**	0.54	0.431	0.469	0.261	3.3	60

**Table 9:** Comparison results.

Model	P%	R%	mAP @0.5/%	mAP @0.5:0.95/%	Parameters /M	FPS
**YOLOv8n**	0.802	0.758	0.807	0.562	3.0	61
**YOLOv12n**	0.811	0.76	0.818	0.572	2.6	65
**RTDETR**	0.787	0.768	0.825	0.581	32.0	20
**SSH-YOLO**	0.847	0.834	0.868	0.648	3.3	60

#### COCO small target and intensive scene experiment.

#### CityPersons occlusion scene experiment.

#### BDD100K experiments in complex environments.

#### KITTI complex environment experiment.

### Generalization performance analysis

**Fig 16:**
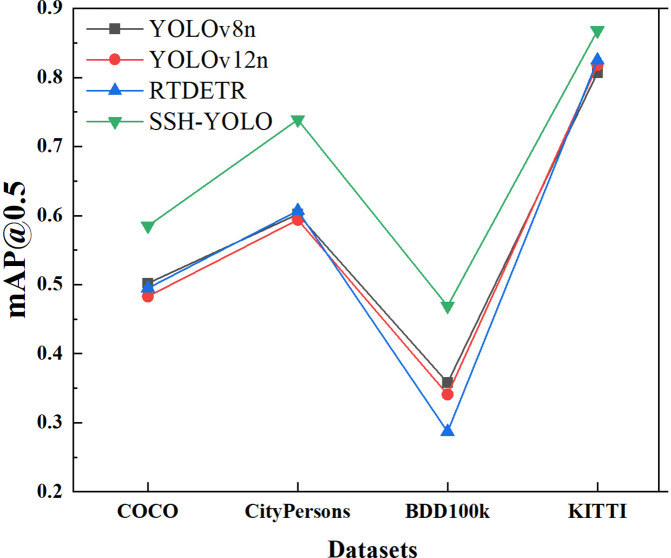
mAP@0.5.

**Fig 17:**
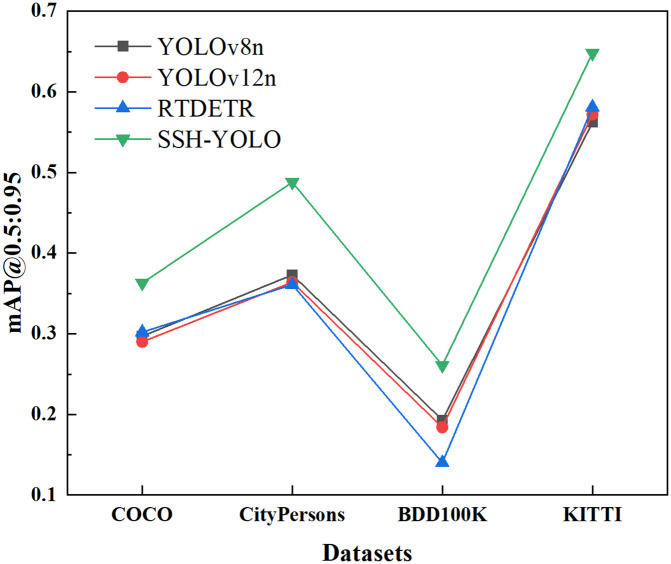
mAP@0.5:0.95.

Figs 16 and 17 correspond to the experimental results of the four public datasets in Chapter 5.2. The name of the dataset is annotated on the abscissa, and the index value is displayed on the ordinate, which intuitively reflects the generalization ability of each model in typical road scenarios such as small targets, dense targets, occlusion scenes, and complex environments.

From this, the following conclusions are drawn:

1) All-scene leadership: The SSH-YOLO mAP@0.5 and mAP@0.5:0.95 on the four datasets were significantly higher than those of the other models, especially in the CityPersons occlusion scene (0.). 739) and the KITTI complex environment (0.). 868), which were 13.2% and 5.0% higher than those of the suboptimal models (RTDETR and YOLOv12n), respectively. This shows that its multilevel feature fusion architecture (SPDConv+SCSA+small target detection head) has a universal optimization effect for complex scenes.
2) Difference in scene sensitivity: In the BDD100K complex environment dataset (including lighting and weather changes), the mAP@0.5 of SSH-YOLO is 0469, which is higher than that of YOLOv8n (35.8%), but the improvement is smaller than that of occlusion or small target scenes. This suggests that there is still room for optimization of the model’s robustness to light and weather interference, and it needs to be further improved in combination with domain adaptation technology.
3) Lightweight and performance balance: Although the number of parameters in the YOLOv12n and YOLOv5n models is lower, SSH-YOLO achieves the best cost-effectiveness in terms of the precision‒parameter trade-off because of its efficient attention mechanism and structured convolution, which verifies the engineering practicability of its architecture design.

## Conclusions

To address the three major pain points of the lightweight YOLO model in complex road scenarios, i.e., feature dilution, insufficient occlusion robustness, and small target detection blind spots, this paper innovatively integrates the SPDConv module, SCSA attention mechanism and four-level detection architecture to construct the SSH-YOLO model, and its innovation and experimental effects are as follows:

Core innovative design: Design the SPDConv module of “nonstep convolution + channel splitting and reorganization”: retains high-resolution features without adding redundant parameters to solve the core contradiction between “lightweight” and “feature retention”. The SCSA attention mechanism of “multiscale volume branching + channel-space dual-domain weight dynamic allocation” is proposed: it is different from single-dimensional attention (such as SE and SAM) and simultaneously realizes the enhancement of small target features and occlusion interference suppression, which fills the robustness gap of the lightweight model in complex occlusion scenarios. A “P2-P5” four-level detection architecture is constructed: a new 160 × 160 resolution P2 detection layer is combined with the bottom-up feature supplementary path (the P2 layer transfers fine-grained texture features to the P3-P5 layers), “10-200 pixels” full-scale road target coverage in the 3 M lightweight model is realized for the first time, and the problem of long-distance small target detection blind spots is solved.

Experimental effect verification: The effectiveness of the innovative design is verified via an ablation experiment and a multidataset comparison experiment, and the core results are as follows:

1) SPDConv alone effect: Model mAP@0.5 increased by 12.4 percentage points to 0.723 compared with the YOLOv8n baseline (0.599), with an AP increase of 18.7% for occluded targets (e.g., semioccluded vehicles) and 18.7% for small targets (<32 × 32 pixels). The AP increased by 21.3%, confirming its fine-grained feature retention ability. 2) Synergy between the SCSA and SPDConv: When the two are used together, mAP@0.5 is further increased to 0.729 (an increase of 0.6 percentage points compared with SPDConv alone), and the missed detection rate of occlusion scenes is reduced by 29.4%, achieving a synergistic gain of “1 + 1>2”.
3) Four-level detection architecture for small target performance: After adding the P2 layer, the AP of the bicycle category increased by 65.2%, from 0.290 to 0.479, and the missed detection rate of ultrasmall targets with < 32 × 32 pixels decreased from 38.2% to 16.5%, effectively covering the blind spot of small target detection.
4) Balance between lightweight and high performance: On RoadScene-Complex and four public datasets, SSH-YOLO leads the overall index with 3.3 M parameters (only 29.5% of YOLOv8n and 1.3% of YOLOv4), with an average mAP@0 of 0.732 (13.3 percentage points higher than YOLOv8n) and an inference speed of 60 FPS, achieving the best cost-effectiveness in the accuracy‒parameter trade-off.

In summary, SSH-YOLO targets the core pain points of lightweight models and provides a “real-time, lightweight, and high-robustness” detection solution for autonomous driving vehicle edge devices.
